# Assessment of Water Quality and Identification of Polluted Risky Regions Based on Field Observations & GIS in the Honghe River Watershed, China

**DOI:** 10.1371/journal.pone.0119130

**Published:** 2015-03-13

**Authors:** Chang-An Yan, Wanchang Zhang, Zhijie Zhang, Yuanmin Liu, Cai Deng, Ning Nie

**Affiliations:** 1 State Key Laboratory of Pollution Control & Resource Reuse, School of the Environment, Nanjing University, Nanjing 210093, P. R. China; 2 Kunming Institute of Environmental Science, Kunming 650032, P.R. China; 3 Key Laboratory of Digital Earth Science, Institute of Remote Sensing and Digital Earth (RADI), Chinese Academy of Sciences (CAS), Beijing 100094, P. R. China; 4 College of Automation, Nanjing University of Posts and Telecommunications, Nanjing 210093, P.R. China; CAS, CHINA

## Abstract

Water quality assessment at the watershed scale requires not only an investigation of water pollution and the recognition of main pollution factors, but also the identification of polluted risky regions resulted in polluted surrounding river sections. To realize this objective, we collected water samplings from 67 sampling sites in the Honghe River watershed of China with Grid GIS method to analyze six parameters including dissolved oxygen (DO), ammonia nitrogen (NH_3_-N), nitrate nitrogen (NO_3_-N), nitrite nitrogen (NO_2_-N), total nitrogen (TN) and total phosphorus (TP). Single factor pollution index and comprehensive pollution index were adopted to explore main water pollutants and evaluate water quality pollution level. Based on two evaluate methods, Geo-statistical analysis and Geographical Information System (GIS) were used to visualize the spatial pollution characteristics and identifying potential polluted risky regions. The results indicated that the general water quality in the watershed has been exposed to various pollutants, in which TP, NO_2_-N and TN were the main pollutants and seriously exceeded the standard of Category III. The zones of TP, TN, DO, NO_2_-N and NH_3_-N pollution covered 99.07%, 62.22%, 59.72%, 37.34% and 13.82% of the watershed respectively, and they were from medium to serious polluted. 83.27% of the watershed in total was polluted by comprehensive pollutants. These conclusions may provide useful and effective information for watershed water pollution control and management.

## Introduction

With the rapid economic and social development in recent decades, non-point source pollution to the environment from livestock and poultry industry, aquaculture industry, planting industry, and rural domestic sewage to our living space centered on the Earth has drawn much attention to the public and policy-makers. Among various pollutions, water environmental pollution, as a vital threat to human being health, also became the most remarkable issue for the sustainable development. Niemi GJ et al [1] reported that human activities mainly impact surface water quality through effluent discharges, using of agricultural chemicals, in addition to the increased exploitation of water resources. Many rivers in the developing countries are heavily polluted due to anthropogenic activities [2], especially in China. There are 426 of 532 rivers under monitoring that are undergoing different kinds and levels of pollutions, and 13 river sections of 7 main rivers in China flowing through 15 cities are highly polluted [3]. According to “Annual Report of Environment Quality in China, 2011” [4], Yangtze River and Zhujiang River were in good condition, Songhua River and Huaihe River were lightly polluted, Yellow River and Liaohe River were in medium contaminated, while Haihe River was heavily polluted. In general, the water quality monitoring for 204 rivers in 409 national river sections indicated that I-III, IV-V and poor V accounted for 59.9%、23.7% and 16.4%, respectively. The water pollution in China has become a serious issue to economic, social sustainable development, not only because the imbalance between available scant water resource and dense population, but also the inefficient of water resources regulation and management. As the secondary tributary on the upper reaches of the Huaihe River, the biggest river in the eastern China, the water quality of the Honghe River will definitely affect the Huai River. It is, therefore, essential to investigate and assess the present situation of water pollution along the Honghe River in the Honghe watershed, so as to understand the whole conditions of the Huaihe River Basin in Eastern China.

Water quality evaluation is considered as critical issue in recent years, especially when freshwater is becoming a scarce resource in the future [5]; the world-widely used principal methods for water quality assessment include single factor pollution index (SFPI) [6], complex pollution indices (CPI) [7], analytic hierarchy process (AHP) [8], fuzzy comprehensive evaluation (FCE) [9], gray evaluation (GE) [10], artificial neural network (ANN) [11], principal component analysis (PCA) [12], Fuzzy comprehensive-quantifying assessment (FCQA) [13], water quality identification (WQI) [14, 15]…etc. However, these methods have a common disadvantage: they have to work with the spatial discontinuity of sampling data. This disadvantage directly leads to an obvious shortcoming of such methodology that they cannot identify hazardous and vulnerable regions resulted from polluted surrounding river sections. Water quality assessment at the basin scale requires not only a large number of variable and corresponding evaluation factors, but also a spatial distribution of pollution levels based on every variable and evaluation factor. GIS, as the most powerful tool for handling spatial data, performing spatial analysis and manipulating spatial outputs [16], becomes a unique tool for geo-statistical analysis and spatial interpolation utilizing measured samples with known values to estimate unknown values so as to visualize the pollution spatial patterns [17]. GIS and modeling have been specifically used in risk assessment and environmental pollution studies at a watershed scale [18–25].

Aiming at evaluating water quality spatially and identifying the potential polluted risky zones with GIS approach, this paper deals with the site observation data of water quality collected from a field campaign conducted within about 15 days in the Honghe River watershed located in the upper reaches of the Huaihe River Basin, Eastern China.

## Methods and Materials

### 1 Study area

No specific permits were required for the study area. The location is not privately owned or protected, and the study studies did not involve endangered or protected species.

**Fig 1:**
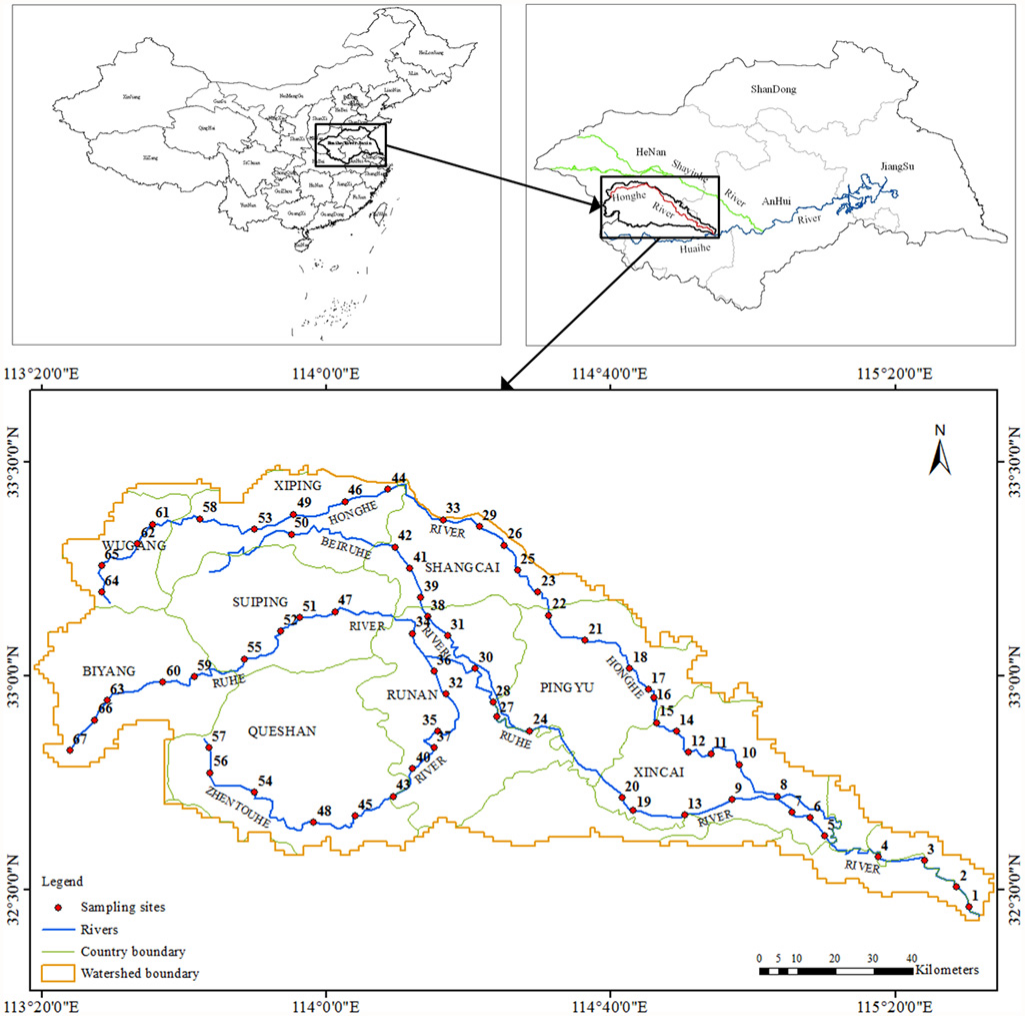
Geographical location and distribution of sampling sites of the Honghe River Watershed.

The study area, Honghe River watershed, is located between N32°25′-33°29′ and E113°19′-115°33′on the up-stream of the Huaihe River Basin, Eastern China (Fig. 1). In Fig. 1, we can see that the Honghe River watershed is situated on the north to Shayinhe River, south to Huaihe main stream, and west to Tangbai River of Yangtze Basin. It flows through Henan and Anhui provinces as well as another thirteen counties (cities) of China. The Honghe River, the secondary tributaries on up-stream of the Huaihe River Basin, originates from mount Nanao in Wugang, Henan province, and drains an extensive area with river channel about 312 km in length. It flows across Wugang city, Wuyang, Xiping, Shangcai, Xincai counties, discharges into the Huaihe River, and three main tributaries, Beiruhe, Zhentouhe, and Ruhe Rivers, constitute its river system. The total drainage of this watershed is about 12,380km^2^, in which mountainous area, hilly area and plain area occupied about 20%, 20% and 60%, respectively.

With rapid economic development in late 1970s, Honghe stream began to experience increased pollution, especially in recent years the surface water of the drainage is polluted dramatically. The contaminants come mainly from domestic sewage, industrial wastewater, agriculture fertilizers, pesticides and human activity productions. The main pollution of the Honghe River watershed is the agricultural non-point source pollution. Enough evidences show that over the last 20 years, water quality in the Honghe River watershed has deteriorated significantly.

### 2 Sampling and Chemical Analysis

In order to ensure enough spatial water sampling representative in such a large watershed while decreasing the pressure of logistic support in the field to the minimum, the sampling strategy was designed to account for enough impacts being posed from the main tributary inputs upon downstream water quality by subdivided the watershed drainage area into 400 equal grids according to geographic location with GIS tool [26]. The sampling activity was conducted following “Technical Specification Requirements for Monitoring of Surface Water and Waste Water” (HJ/T91–2002) [27] in May 2011. Three water samples from each sampling site were taken and analyzed. Each sampling site was positioned by Global Positioning System (GPS) (Table 1), and chemical analyses were carried out immediately after the water samples were brought back, the analyses procedure strictly obeys the guideline described in “Monitoring and Analysis Method of Water and Waste water” [28]. The measured chemical parameters include field DO, NH_3_-N, NO_3_-N, NO_2_-N, TN and TP. All the observed data was facilitated and visualized to perform spatial analysis with GIS software and achieved for further studies.

**Table 1:** The GPS coordinates of the sampling sites.

S	E/°	N/°	S	E/°	N/°	S	E/°	N/°	S	E/°	N/°	S	E/°	N/°
**1**	115.51	32.46	**15**	114.79	32.88	**29**	114.36	33.35	**43**	114.16	32.72	**57**	113.72	32.83
**2**	115.48	32.51	**16**	114.77	32.95	**30**	114.35	33.02	**44**	114.14	33.44	**58**	113.70	33.36
**3**	115.41	32.57	**17**	114.76	32.97	**31**	114.29	33.09	**45**	114.07	32.67	**59**	113.69	33.00
**4**	115.29	32.58	**18**	114.76	32.97	**32**	114.29	32.97	**46**	114.04	33.41	**60**	113.62	32.99
**5**	115.17	32.63	**19**	114.72	32.68	**33**	114.27	33.36	**47**	114.02	33.15	**61**	113.59	33.35
**6**	115.13	32.67	**20**	114.70	32.71	**34**	114.27	33.08	**48**	113.97	32.66	**62**	113.55	33.31
**7**	115.10	32.68	**21**	114.61	33.08	**35**	114.26	32.87	**49**	113.92	33.37	**63**	113.48	32.94
**8**	115.06	32.71	**22**	114.53	33.14	**36**	114.26	33.01	**50**	113.92	33.33	**64**	113.47	33.19
**9**	114.95	32.71	**23**	114.50	33.20	**37**	114.25	32.83	**51**	113.91	33.04	**65**	113.47	33.26
**10**	114.93	32.79	**24**	114.48	32.87	**38**	114.24	33.14	**52**	113.89	33.10	**66**	113.46	32.90
**11**	114.88	32.81	**25**	114.45	33.25	**39**	114.22	33.18	**53**	113.83	33.34	**67**	113.40	32.82
**12**	114.85	32.82	**26**	114.42	33.31	**40**	114.20	32.80	**54**	113.83	32.73			
**13**	114.84	32.67	**27**	114.40	32.91	**41**	114.19	33.25	**55**	113.81	33.04			
**14**	114.82	32.87	**28**	114.40	32.94	**42**	114.16	33.30	**56**	113.72	32.77			

**Note: S: Sampling sites; E: Longitude; N: Latitude**.

### 3 Water quality assessment method

Single factor pollution index method and comprehensive pollution index referred to the level III water quality categories cited in “*Environmental Quality Standards for Surface Water*” (GB3838–2002, GHZB1–1999) [29, 30], published by the State Environment Protection Administration (SEPA) of China, were adopted to assess the water quality of the study area. DO, NH_3_-N, NO_3_-N, NO_2_-N, TP and TN, were selected as the basic criterion through surface water environment function zoning made by GIS for water quality assessment. All mathematical and statistical computations were made by SPSS 13.0. Single factor pollution index method was formulated as:

$$P_{i}=\frac{C_{i}}{S_{i}}$$ (1)

where, *P*_*i*_ refers to the pollution index of *i* units pollutant. *C*_*i*_ refers to the measured concentration of *i* units pollutant (mg/L), *S*_*i*_ the III level water quality standard category value of *i* units pollutant according to “Environmental Quality Standards for Surface Water”. The water quality factors reach the water quality standards as long as *P*_*i*_ ≤1, as the smaller the *P*_*i*_, the better quality the water is. On the contrary, as the water quality factors *P*_*i*_>1, it implies that the water was polluted, with the increase of *P*_*i*_ the heavier polluted the water became. We can use five levels to describe the single factor pollution in the way of single factor pollution index, as the Table 2 listed here below:

**Table 2:** Standard of single factor pollution index.

Pi	≤0.4	0.4~1.0	1.0~2.0	2.0~5.0	>5.0
Pollution levels	Non-pollution	Slight polluted	Medium polluted	Heavy polluted	Serious polluted

As water quality is a complex issue that involved many different kinds of contaminants in surface water, comprehensive pollution index method might be essential for much scientifically reflecting the kinds and level of main pollutions according to water pollution level standards [31]. The comprehensive pollution index method can be formulated as:

$$P=\frac{1}{n}\sum_{i=1}^{n}\frac{C_{i}}{S_{i}}$$ (2)

where, *P* represents comprehensive pollution index, *C*_*i*_ the measured concentration of *i* units pollutant (mg/L), *S*_*i*_ the III level water quality standard category value of *i* units pollutant according to “Environmental Quality Standards for Surface Water”, and *n* is the number of selected pollutants. The values determined for *P*, as listed in Table 3, could be used to classify the water quality level of the surface water at the basin.

**Table 3:** Standard of comprehensive pollution index classification.

P	≤0.2	0.2~0.40	0.40~0.70	0.70~1.0	1.0~2.0	>2.0
WQC	I	II	III	IV	V	Poor V
PL	cleanness	Sub-cleanness	Slight polluted	Medium polluted	Heavy polluted	Serious polluted

**Note: WQC: Water quality classification; PL: Pollution levels**.

## Results and Discussion

### 1 Water Quality Assessment

The water pollution level determined for 67 samples with 6 water quality parameters by single factor pollution index method are shown in Table 4.

**Table 4:** Statistics for the 6 Water Quality Parameters Derived from 67 Samples and Water Quality Assessed by Single Factor Pollution Index Method in the Honghe River Watershed.

WQP	Level III	MS	ESS	AESR/ (%)	AEST	Mean/ (mg/L)	SD	ASPI	Pollution level
DO	≥5	67	31	46.27	0.39	4.97	2.04	0.99	Slight polluted
NH3-N	≤1	67	14	20.90	1.27	0.64	0.97	0.64	Slight polluted
TN	≤1	67	36	53.73	2.59	2.05	2.32	2.05	Heavy polluted
TP	≤0.2	67	63	94.03	6.48	1.42	1.14	7.09	Serious polluted
NO3-N	≤10	67	0	0	0	1.27	1.80	0.26	Non-pollution
NO2-N	≤0.15	67	27	40.30	0.98	0.14	0.15	8.44	Serious polluted

Note: WQP: Water Quality Parameter; MS: Monitoring Section; ESS: Exceeding Standard Section; AESR: Averaged Exceeding Standard Rate; AEST: Averaged Exceeding Standard Times; SD: Standard Deviation; ASPI: Averaged Single Pollution Index.

The data in the Table 4 indicates all the monitoring sections has undergone various pollutions differently, but mostly come from TP and NO_2_-N. According to the level III standard in “*Environmental Quality Standards for Surface Water*”, TP in all 63 monitoring sections all exceeded the standard limit of the category, reaching 94.03% in total, with 6.48 times higher than normal standard. The average pollution index was 7.09 in all monitoring sections, much higher than the limit value of serious polluted level (Table 2). Similarly, the NO_2_-N pollution also reached the serious polluted level with the average pollution index of 8.44, specifically, there were 27 exceeding standard sections in total with 40.30% averaged exceeding standard rate and 0.98 averaged exceeding standard times. TN, compared with phosphorus and NO_2_-N, was in better situation, which had about 36 exceeding standard sections accounting for 53.73% in total, and the exceeding standard times and average single pollution index were 2.59 and 2.05, respectively, the pollution level was classified into heavily polluted. Both of DO and NH_3_-N were in slightly polluted level, with the average pollution index of 0.99 and 0.64, accordingly, 31 and 14 exceeding standard sections took up the averaged exceeding standard times of 0.39 and 1.27, respectively. The concentrations of NO_3_-N in all the monitoring sections were much lower than the standard limit of Category III. As far as what this paper was concerned, the TP, NO_2_-N and TN constituted of the main pollutants were far beyond the standard limit of Category III. Based on studies by Liu [32], it implied that non-point source caused by livestock and poultry industry, aquaculture industry, and planting industry was the major pollution source in Honghe River Watershed.

**Fig 2:**
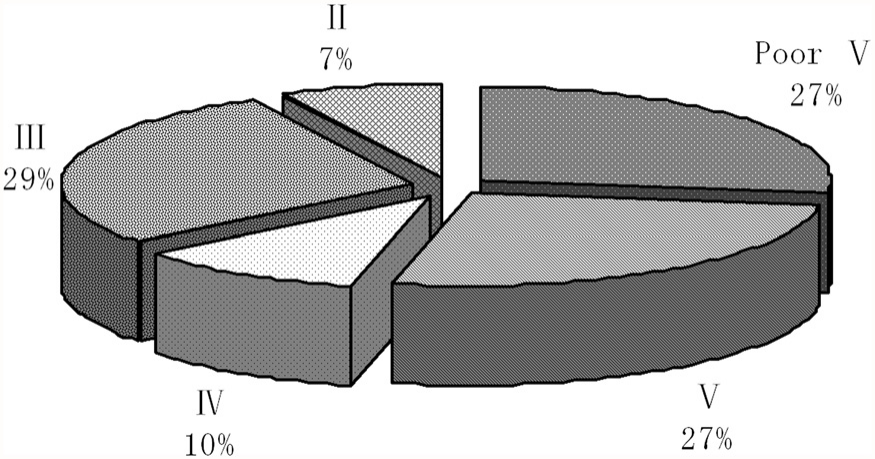
The proportional graph of water quality levels in the Honghe River Watershed.

According to the water quality assessment results obtained by comprehensive pollution index method listed in Table 3, the pie chart in Fig. 2 exhibited the water quality levels. Among 67 monitoring sections, 18 monitoring sections accounting for 26.87% in total were polluted seriously, water quality then was categorized in Poor V. 18 monitoring sections, about 26.87% of all, were heavily polluted, water quality was categorized in level V. 7 monitoring sections occupying about 10.45% in total were medium polluted and categorized in level IV. 19 monitoring sections accounting for 28.36% in total were slightly polluted and categorized in Category III. Only 5 monitoring sections accounting for 7.45% of all were categorized in sub-cleanness, where water quality was classified into level II. In summary, I-III water quality levels only took up 36%, and all the rest belonged to water quality Category IV and higher, therefore, the water quality in the Honghe river watershed was poorer in general.

### 2 Identification of Polluted Risky Regions

In order to characterize the spatial pattern of the polluted and risky vulnerable zones in study area, the spatial distribution of single factor pollution index as well as comprehensive pollution indices for NH_3_-N, NO_2_-N, TP, TN, and DO were processed with GIS, geostatistical methods. Two evaluation criteria such as Mean Standardized Prediction Error (MSPF) and Root Mean Square Standardized Prediction Error (RMSS) were applied to recognize appropriate geostatistical method of spatial interpolation. The closer to 0 for MSPF and 1 for RMSS, the more precision it is. According to experimental results of different geostatistical methods of spatial interpolation, the most efficient and prominent method for observed data was the Ordinary Kriging (OrKrig) [33]. These evaluate results which were obtained by OrKrig interpolation method are best and acceptable, as the Table 5 shows here below:

**Table 5:** Results of the evaluation criterions of spatial interpolation method for single factor pollution index and comprehensive pollution indices.

Object of spatial interpolation	Method of spatial interpolation	MSPE	RMSS
single factor pollution index of NH_3_-N	OrKrig	0.02982	0.8730
single factor pollution index of NO_2_-N	OrKrig	0.00650	0.9327
single factor pollution index of TP	OrKrig	0.02378	0.9309
single factor pollution index of TN	OrKrig	0.00435	0.9942
single factor pollution index of DO	OrKrig	-0.02169	1.0220
comprehensive pollution indices	OrKrig	0.02623	0.8992

**Fig 3:**
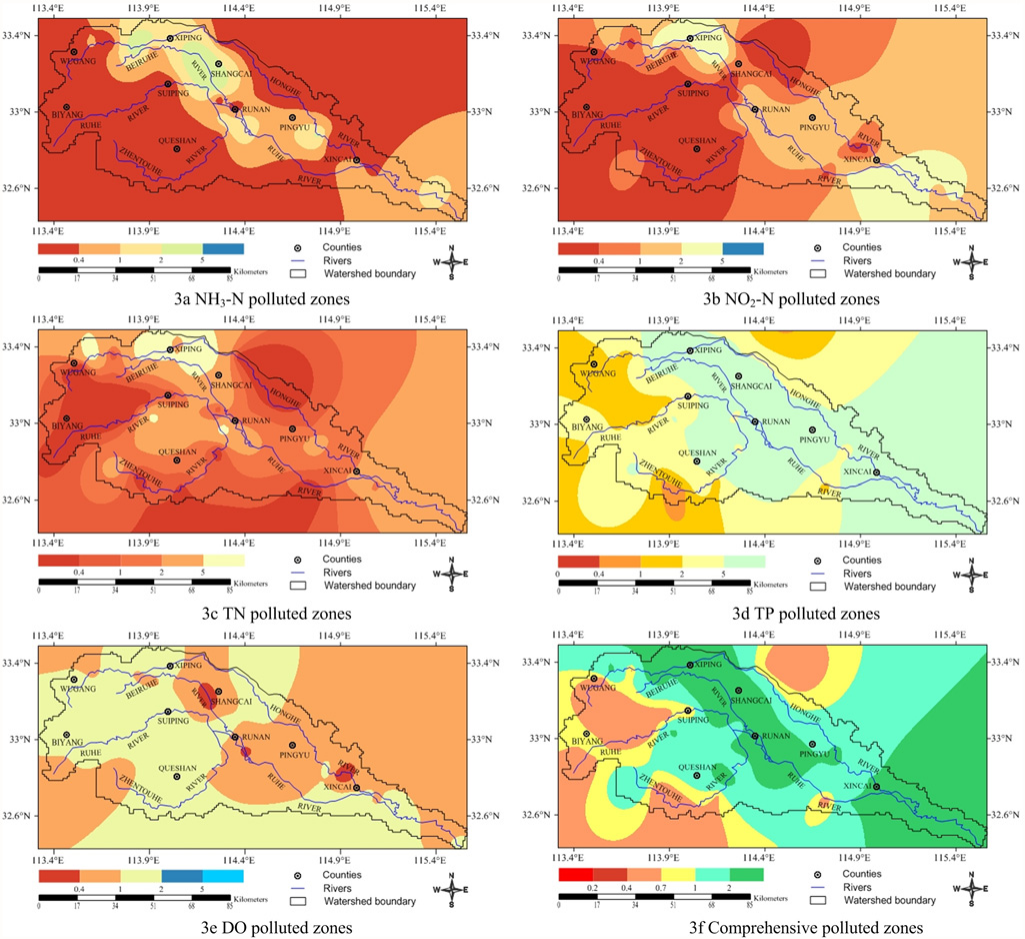
The spatial pattern of the polluted and risky vulnerable zones across the Honghe River Watershed based on pollution index.

Fig. 3a illustrated the spatial variability of the single factor pollution index of NH_3_-N. From these maps, the dispersion of NH_3_-N pollution in the study area can be recognized and five major zones with NH_3_-N pollution over the watershed can be found. The first zone, defined as non-pollution area, located surrounding the Wugang, Queshan and Suiping counties, almost covered more than half of the study area (62.14%) as indicated in Table 6. The second zone, defined as slightly polluted area, mainly concentrated in Zhentouhe and Honghe tributary river sections, covered about 24.04% of the study area. The third zone, defined as moderately polluted area, were separated one from another by miles of open land to the northwest, north-central, east and middle parts of the study area, covered 8.62% of the study area. The fourth zone was the heavily polluted area, covering only 5.2% and mainly distributed in the northwest part of the study area. The fifth zone, defined as seriously polluted area, was almost 0% in study area. So far, most parts of the watershed were polluted lightly by NH_3_-N, and the rest was under medium and heavy pollution risk.

**Table 6:** GIS extracted coverage percentage of the study area for NH_3_-N, NO_2_-N, TN, TP and DO.

Parameter	Percentage of classes (%)				
	0–0.4	0.4–1	1–2	2–5	>5
NH3-N	62.14	24.04	8.62	5.20	0
NO2-N	42.86	19.80	26.55	10.79	0
TN	18.51	19.27	26.01	29.96	6.25
TP	0	0.93	13.75	27.62	57.70
DO	1.83	38.45	59.72	0	0

Concerning the single factor pollution index of NO_2_-N and the single factor pollution index of NO_3_-N, the spatial characteristics over the study area were interpolated with OrKrig interpolation method, and the result was shown in Fig. 3b. Five categories regarding to pollution levels were classified non-pollution, slightly polluted, moderately polluted, heavily polluted and seriously polluted to evaluate the spatial characteristics of each pollutant over the studied watershed. As summarized in Table 6, non-pollution zone was about 42.86%, slightly polluted zone occupied about 19.80%, the moderately polluted zone took up 26.55% of the whole watershed, respectively, and mainly of them located in the northwest, middle, and easternmost parts of study area. The last 10.79% of the study area, distributed in the northwest and east part of the study area, was defined as the heavily polluted zone. Serious polluted zone based on this analysis didn’t appear up.

Fig. 3c exhibited the spatial characteristics of TN pollution and the classified pollution levels, obtained by the similar way as NO_2_-N. As Table 6 list below, the non-pollution, slightly polluted, moderately polluted, heavily polluted and seriously polluted zones were accounting for about 18.51%, 19.27%, 26.01%, 29.96% and 6.25% of the whole watershed, respectively. The pollution above the moderate polluted level predominated most of the watershed areas except the westernmost, south-central and north-central parts of the watershed.

The spatial distribution of TP pollution referred by single factor pollution index is displaying in Fig. 3d. According to the spatial analyses on TP pollution levels in Fig. 3d and the water parameters coverage percentage in Table 6, about 57.7% of the watershed, mainly located in the northwest, was seriously polluted. Middle and south, approximately covered 27.62% and 13.75% of the watershed were heavily and moderately polluted. Only about 0.93% of the watershed was recognized as slightly polluted area. To wrap it up, the whole watershed was seriously polluted by the TP.

The TP pollution spatially interpolated with OrKrig approach for DO was mapped, spatial distributions for five pollution levels processed in similar way was exhibited in Fig. 3e. Spatial analyses on the results shown in Table 6 indicated that most of the watershed was moderately polluted accounting for about 59.72% of the total area, mainly distributed in the Midwest part of the watershed. The rest parts about 1.83% and 38.45% were non-pollution and slightly polluted zone, respectively.

Comprehensive pollution indices for each sampling site was spatially interpolated with OrKrig approach, and it was classified into 6 pollution levels according to classification standards on comprehensive pollution indices. The results were presented in Fig. 3f. Based on the statistics, the spatial characteristics of the 6 pollution levels were analyzed, and the following conclusions were drawed: (1) The cleanness level with comprehensive pollution indices less than 0.2, was almost non-existed in the studied watershed. (2) The sub-cleanness level with the comprehensive pollution indices varies from 0.2 to 0.4 only accounted for about 0.01% of the studied area. (3) The slightly polluted level with the comprehensive pollution indices varies from 0.4 to 0.7 covered about 16.72% of the studied area. (4) The moderately polluted level with the comprehensive pollution indices varies from 0.7 to 1.0 occupied about 13.69% of the studied area. (5) The heavily polluted level with the comprehensive pollution indices varies from 1.0 to 2.0 covered 42% of the studied area. (6) The seriously polluted level with the comprehensive pollution indices bigger than 2.0 took up about 27.58% of the rest. Those heavily even seriously polluted areas mainly located in the northeast, middle, south-central and easternmost parts of the watershed.

**Fig 4:**
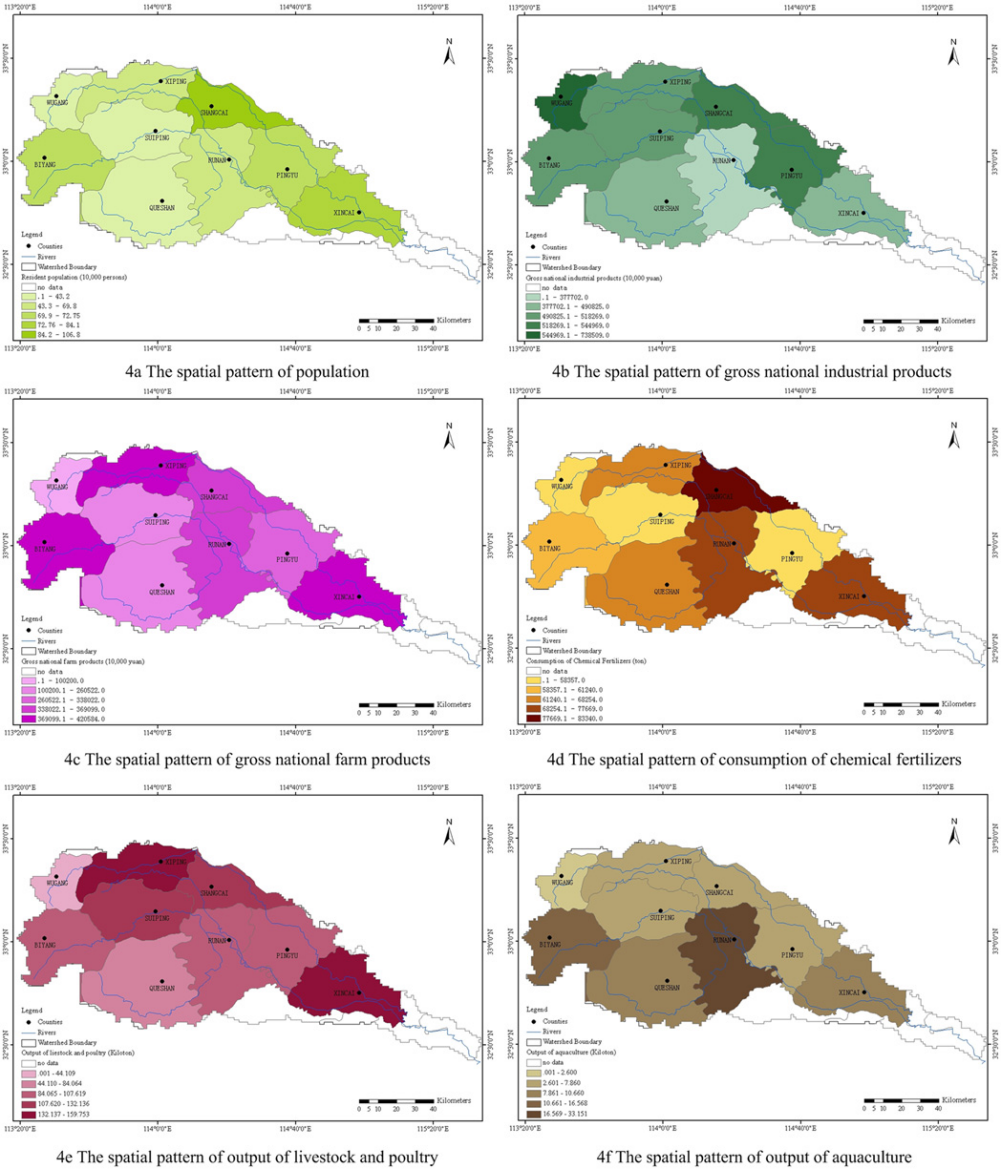
The spatial pattern of the major social-economic statistical indicators at county level of the Honghe River Watershed in 2011.

Liu [32] showed us that the pollution of surface water in Honghe River Watershed was mainly the agricultural non-point source pollution. It mainly included pollutions from livestock and poultry industry, aquaculture industry, planting industry, and rural domestic sewage. Six spatial patterns of related social-economic statistical indicators at county level of the Honghe River Watershed in 2011 [34] were used to verify reliability of the identified pollution risky regions, which were shown in Fig. 4. Specifically, Fig. 3a displayed that Shangcai Area was most polluted by NH_3_-N, and the possible reasons for this were the largest population (Fig. 4a) and the largest consumption of chemical fertilizers (Fig. 4d). Likewise, the output of livestock and poultry in Xiping County (Fig. 4e) powerfully proved that this area was most polluted by TN (Fig. 3c). In other words, domestic sewage, livestock and poultry industry were the mainly polluted sources of NH_3_-N and TN in Honghe River Watershed. All these inferences had been confirmed by Liu [32].

## Conclusions

Therefore, in this paper, the evaluation method combining with single factor pollution index, comprehensive pollution index and GIS approach was successfully applied to evaluate water pollution variability of major water pollutants at monitoring sites and identify the potential polluted risky zones in Honghe River Watershed, upper stream of the Huaihe River Basin, eastern China. The results indicated: referring to the value standardized by Category III in “*Environmental Quality Standards for Surface Water*”, TP, NO_2_-N and TN were the main and serious excessive pollutants. According to the classification standards of pollution index, the whole water quality was comparatively poorer, with 22% of sections in Category I-III and 68% in Category IV-poor V. The main reasons to the watershed pollution were the discharge of industrial and agricultural wastes, domestic sewage such as people and livestock excrements around the watershed.

Geostatistical analysis and GIS helped to identify the polluted risky regions for each parameter. The zones of TP, TN, DO, NO_2_-N and NH_3_-N pollution, covering 99.07%, 62.22%, 59.72%, 37.34% and 13.82% of the watershed respectively and undergoing from medium to serious pollution, mainly distributed in northwest and middle of the watershed, and must be paid highly attention by water quality management department. Similarly, 83.27% of the watershed in total was polluted by comprehensive pollutants of medium, heavy and serious polluted level, which mainly lied on the northeast, middle, south-central and easternmost of the watershed. At the end of this paper, combined with spatial patterns of social-economic statistical indicators at county level of the Honghe River Watershed in 2011, this paper analyzed the major source of water pollution in Honghe River Watershed and verified the reliability of the identified polluted risky regions.

It is believed that these reliable results could be very useful and valuable to pollution control strategies, as well as future plan and management on the watershed; besides, they are also helpful to further research on water quality simulation and validate the simulation accuracy in watershed space.
